# Chronic recurrent multifocal osteomyelitis. A narrative and pictorial review

**DOI:** 10.3389/fimmu.2022.959575

**Published:** 2022-08-22

**Authors:** Consolato M. Sergi, Elka Miller, Dina El Demellawy, Fan Shen, Mingyong Zhang

**Affiliations:** ^1^ Anatomic Pathology Division, Children’s Hospital of Eastern Ontario (CHEO), Ottawa, ON, Canada; ^2^ Department of Laboratory Medicine and Pathology, University of Alberta, Edmonton, AB, Canada; ^3^ Department of Orthopedics, Tianyou Hospital, Wuhan University of Science and Technology, Wuhan, China; ^4^ Medical Imaging Department, Children’s Hospital of Eastern Ontario (CHEO), University of Ottawa, Ottawa, ON, Canada

**Keywords:** osteomyelitis, chronicity, multilaterality, autoinflammatory, bone

## Abstract

Chronic recurrent and multifocal osteomyelitis (CRMO) is a nonsporadic autoinflammatory disorder. Currently, it is diagnosed based on clinical, radiologic, pathological, and longitudinal data. Numerous aspects should be highlighted due to increased knowledge in imaging and immunology. We emphasize the use of whole-body MRI, which is a non-invasive diagnostic strategy. A literature review was carried out on longitudinal studies. Commonly, the mean age at diagnosis is 11 years, ranging between 3 and 17. The most common sites are the long bone metaphysis, particularly femoral and tibial metaphysis. In addition, the pelvis, spine, clavicle, and mandible may be involved. In long bones, the radiologic appearance can show typical structure, mixed lytic and sclerotic, sclerotic or lytic. It is frequently metaphyseal or juxta-physeal, with hyperostosis or periosteal thickening. The involvement of the vertebral skeleton is often multifocal. Therefore, whole-body MRI is essential in identifying subclinical lesions. CRMO is a polymorphic disorder in which whole-body MRI is beneficial to demonstrate subclinical edema. Vertebral collapse requires long-term monitoring.

## Introduction

Osteomyelitis is a common condition that involves the bone marrow. It may present with acute and chronic features and variable etiology. In most cases, acute osteomyelitis is due to hematogenous spread. Microorganisms can enter a bone in various ways, including in the bloodstream. Therefore, acute hematogenous osteomyelitis is a common diagnosis. It is often takes place after an episode of bacteremia in which the bacteria inoculate the bony tissue. The microorganisms frequently involved are *Staphylococcus aureus*, *Streptococcus pneumoniae*, and *Haemophilus influenzae* type b (1, 2).

On the other hand, chronic recurrent multifocal osteomyelitis (CRMO) is an inflammatory bone disorder lacking bacterial involvement. It is characterized by lytic, sclerotic, and hyperostotic lesions. Despite the chronicity character, CRMO often exhibits periodic flairs and phases of remission (3). CRMO is currently indicated as an autoinflammatory disease. It often affects the pediatric population, and the onset age is usually nine years, with a female to male ratio of 2:1. CRMO is occasionally labeled SAPHO, an acronym describing these patients’ clinical features, including synovitis, acne, pustulosis, hyperostosis, and osteitis syndrome. Another name, which some rheumatologists also use, is chronic nonbacterial osteomyelitis (CNO), or simply nonbacterial osteomyelitis (4, 5). It has been suggested that SAPHO is often nonbacterial osteomyelitis with an autoinflammatory character of the adult, while CRMO should be limited to the pediatric age group (6–13).

## Etio-pathogenesis

There is a substantial body of evidence that rejects any infection or colonization in CRMO, and despite some treatments, there is no positive result apart from single or anecdotal improvements in patients receiving macrolide therapy. Macrolides are also known to harbor anti-inflammatory activity (14, 15). It has been clarified that the anti-inflammatory properties of macrolides are linked to the chemical structure. Immunomodulatory effects have been detected with 14- (erythromycin, clarithromycin and roxithromycin) and 15- (azithromycin). No results with 16-member (josamycin) macrolides have been identified. The most frequently and consistently reported immunomodulatory effect of macrolides is decreased neutrophilic inflammation. A decrease of neutrophils and inhibition of neutrophilic function lead to lower IL-8 and neutrophil elastase rates, ultimately limiting tissue injury.

Similar to other autoimmune conditions, there is a remarkable imbalance of cytokines in patients affected by CRMO. It has been underlined that patients suffering from CRMO show a decreased production of anti-inflammatory cytokines (interleukins 9, 10, and 18; IL-9, IL-10, IL-18) and increased production of proinflammatory cytokines (IL-1b, IL-6, tumor necrosis factor-α, TNF-a) (4, 16–22). The cytokine imbalance is probably the basis of the bony manifestations of CRMO. In a mouse model, bone, cartilage, and skin inflammation of the extremities and ears have been described (23). These authors detected an L98P mutation in the *Pstpip2* gene and commented that this genetic variation may be the cause of the autoinflammatory phenotype. Lukens et al. revised the critical role of the inflammasome in osteomyelitis (24) and showed that the bone disease in CRMO rodents may harbor NOD-, LRR- and pyrin domain-containing protein 3 (NLRP3). It is inflammasome dependent and, of course, IL-1b mediated (4, 13, 17, 25–32). In some patients affected by CRMO, single-gene defects have been identified. Clinical geneticists have also identified some similarities with Majeed syndrome, which is extremely rare, being described in only 24 individuals from 10 families, and is characterized by recurrent episodes of fever and osteomyelitis, and dermatitis (33, 34).

*LPIN2* (Lipin 2) is a Protein Coding gene. LPIN2 acts as a phosphatidate phosphatase enzyme, magnesium-dependent. The ultrastructural and biochemical investigations revealed that the reticulum endoplasmic membrane is critical. It catalyzes the conversion of phosphatidic acid to diacylglycerol during triglyceride, phosphatidylcholine, and phosphatidylethanolamine biosynthesis. LPIN2 is crucial to controlling fatty acid metabolism and is a nuclear transcriptional coactivator for PPARGC1A to modulate lipid metabolism. This gene seems to be a candidate gene for human lipodystrophy, characterized by fatty liver, body fat loss, hypertriglyceridemia, and insulin resistance. CRMO patients have been demonstrated to have *LPIN2* gene mutations (35).

There is also DIRA, or deficiency of IL-1 Receptor Antagonist, which is a genetic disorder with an autosomal recessive pattern of inheritance and has autoinflammatory clinic-pathological characteristics caused by mutations in the *IL1RN* gene (36). These patients show a pustular rash and nonbacterial osteomyelitis within the first few postnatal weeks. It has been demonstrated that the IL1RN gene instructs for the codification of the interleukin 1 receptor antagonist, which is key in leading to unchecked inflammation. Another gene has been controversially discussed to be an integral part of CRMO. The Filamin-binding LIM protein 1 (*FBLIM1*) gene harboring bi-allelic variants has been described in CRMO patients, although recently, a new study seems to vindicate this gene in causing CRMO. FBLIM1 gene encodes filamin binding lin-11, islet-1, mec-3 (LIM) protein (37, 38). More recently, Yahara et al. (39) have indicated that the interaction between some haplotypes of killer cell immunoglobulin-like receptors and the human leukocyte antigen (HLA) may be critical. This interaction would be able to determine the HLA instability leading inexorably to autoimmunity in CRMO.

## Diagnosis

The approach to CRMO is not a straightforward process because no specific biomarkers are available (17, 18, 40–45). Therefore, we need to rule out malignancies first, then infections and other inflammatory conditions. Thus, expertise in radio-diagnostics and pathology is key in ruling out osteosarcoma and Ewing sarcoma as primary bone tumors, metastases as secondary bony tumors, and leukemia or lymphoma in the setting of a generalized lymphoproliferative disorder (6, 46). Currently, there are two sets of diagnostic criteria, the Jansson and Bristol criteria (47, 48). According to the Jansson system, CRMO is validated by two major, or one major plus three minor criteria (49). In this system, major criteria are as follows: (1) Radiologically demonstrated osteolytic/osteosclerotic bony lesions, (2) Multifocality of the bony lesions, (3) Palmoplantar dermatosis (pustulosis psoriasis), and (4) Sterile osseous biopsy with signs of inflammation, fibrosis, or sclerosis. In the Jansson system, minor criteria include (1) Normal blood count and good general state of health, (2) Increase of C reactive protein (CRP) and erythrocyte sedimentation rate (ESR) (mild to moderate levels), (3) Time for more than half a year, (4) Hyperostosis, and (5) Positive family history with grade I or II relatives diagnosed by any autoimmune disease. In the Bristol system, diagnostic criteria include typical clinical findings (bony pain localized or swelling features of inflammation), typical radiological findings, and one of the following criteria: (1) >1 bone affected without significantly raised CRP (<30 mg/l), (2) Bony biopsy demonstrating inflammation with the recruitment of both chronic inflammatory cells (plasma cells) as well as osteoclasts and histologic evidence of fibrosis or sclerosis with no growth of microorganisms in a status without antibiotic therapy.

## Clinico-laboratory data

In the clinical presentation, some features seem to be recurrent. They include painful inflammatory bony lesions that may affect any skeleton area. The analysis of several studies identified that the more often active regions include the metaphysis of long bones, the pelvis, and the shoulder girdle. Also, the clavicle, vertebral bodies, and lower jaw are often involved. The patients may present to the emergency department with vertebral body fracture, and a skilled radiologist should raise the differential diagnosis properly. Local inflammation may often be identified in the affected extremities (48, 50). Children experience pain, which is worse at night. In about half of the patients reported in the literature, symmetrical involvement of bony lesions is seen. The pain is usually insidious in origin and persists or is intermittent and can last for several years. The multilateral and multifocal aspects are often seen and need to be distinguished by the occurrence of juvenile rheumatoid arthritis (6, 51). Extraosseous features of CRMO include skin, gastrointestinal, ocular, and cardiac involvement. The pustular rash on the palms and soles is quite characteristic (50). Prognosis may be variable, but poor factors include prolonged time from the onset of symptoms to the final diagnosis, multifocality, and male gender (52). Laboratory tests may exhibit a mild to moderate increase of inflammatory markers (50, 53, 54), but lactate dehydrogenase, uric acid, or alkaline phosphatase may be abnormal. Importantly, low albumin may indicate a simultaneous inflammatory bowel disease. In a few patients, there have been reports of autoantibodies or HLA B27.

## Imaging

**Figure 1:**
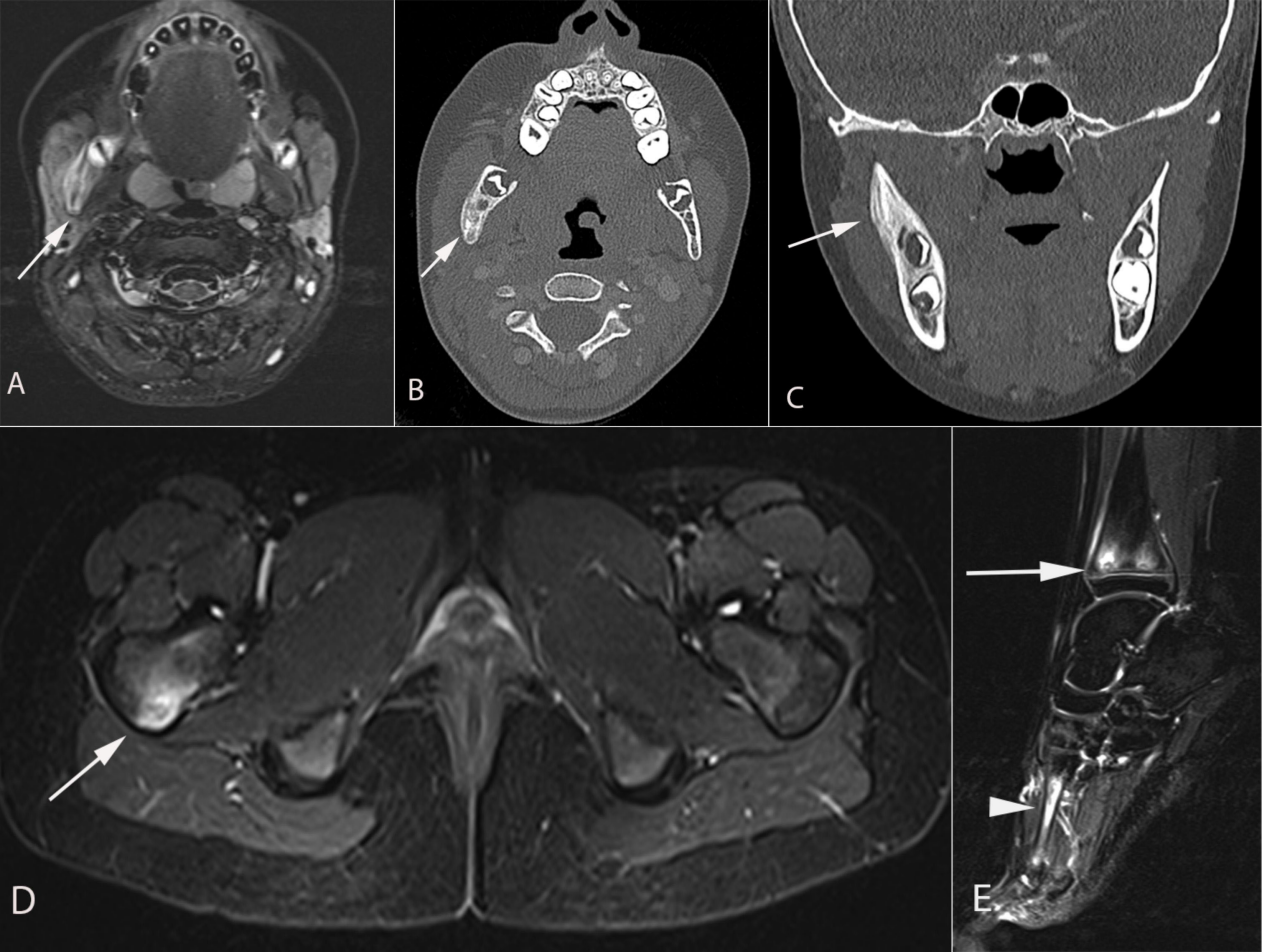
A 9-year-old girl presented with 10 days of right cheek swelling. **(A)** MRI Axial T2 STIR demonstrated asymmetric enlargement of the right masseter muscle with a heterogeneous hyperintense signal of the right mandibular ramus (arrow) in keeping with bone marrow edema. **(B)** axial computed tomography and **(C)** coronal computed tomography images demonstrate smooth periosteal reaction in the right hemi mandible. No associated soft tissue mass or periodontal abscess. Axial **(D)** and sagittal **(E)** MRI STIR images demonstrate another focus of hyperintensity in the posterior aspect of the right femoral neck (arrow in **D**), the distal tibial metaphyses (arrow in e), and the right first (arrowhead in e) and second metatarsals (not shown). Findings are consistent with CRMO following a Whole Body MRI.

**Figure 2:**
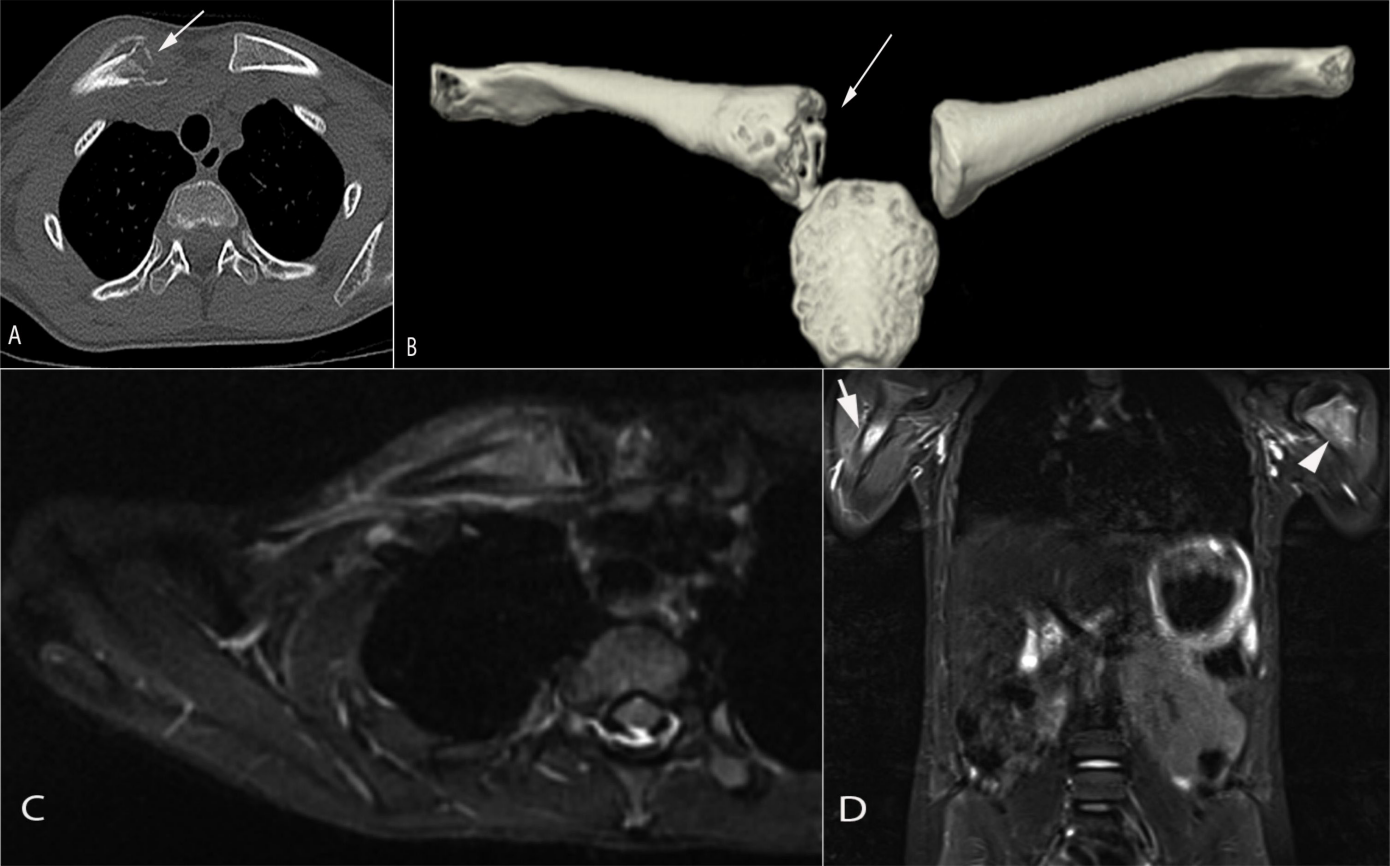
An 8-year-old girl presented with a painful lump on the right clavicle. Axial **(A)** and 3D reformats **(B)** computed tomography images demonstrate a destructive lesion in the medial aspect of the right clavicle associated with periosteal reaction and soft tissue swelling (arrow in a/b). Axial T2 STIR magnetic resonance image **(C)** showed a marked signal abnormality involving the medial aspect of the right clavicle with associated periosteal fluid and soft tissue edema. Total body MRI-STIR sequences **(D)** showed additional lesions in the proximal right (arrow) and left (arrowhead) humeral metaphysis, right iliac crest (not shown)and left greater trochanter (not shown). Findings are consistent with CRMO following a Whole Body MRI.

**Figure 3:**
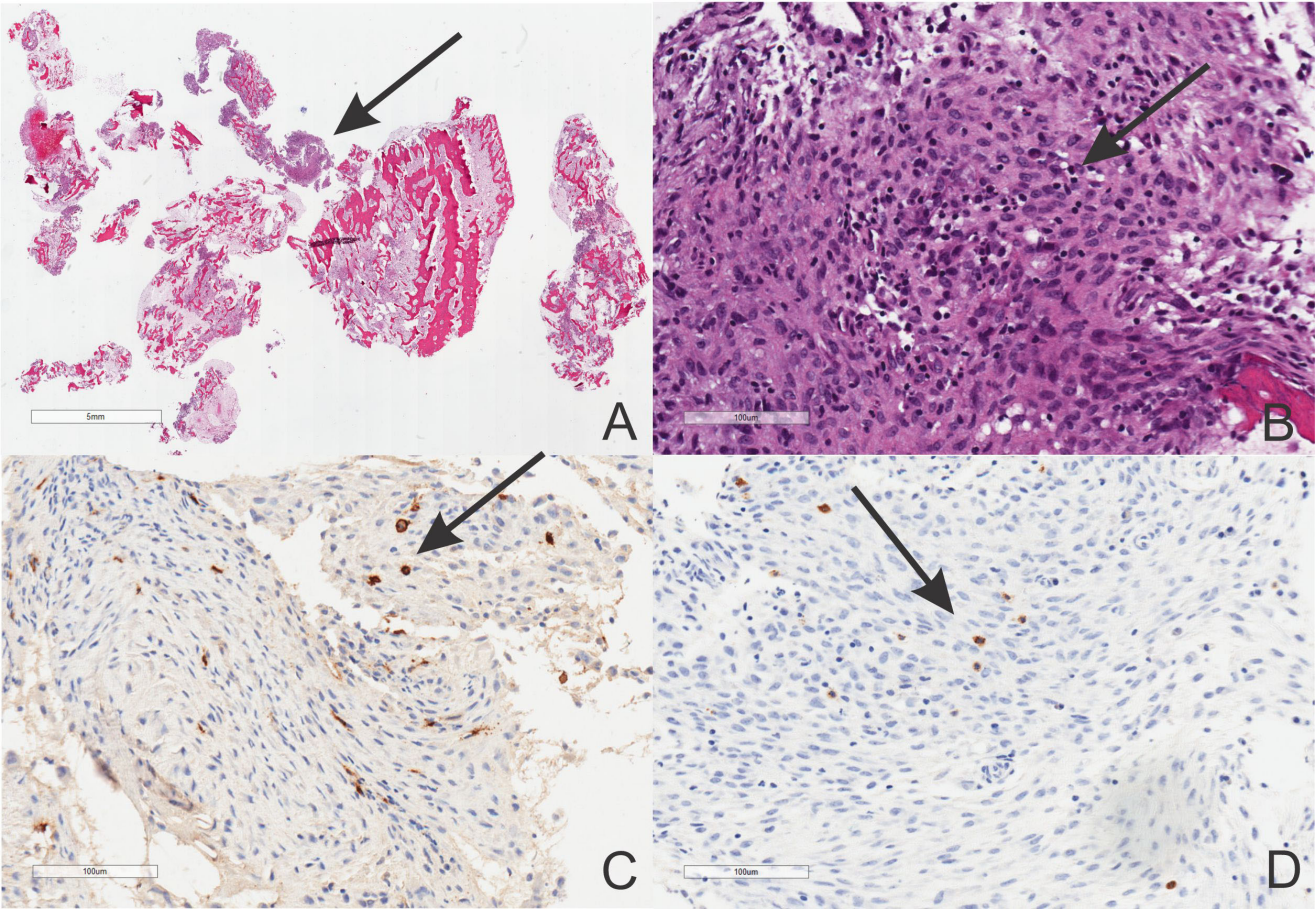
Histopathology of the CRMO showing low and high power magnification of a nonbacterial osteomyelitis (**A, B**, hematoxylin-eosin staining, scale bars included) with CD117 **(C)** and tryptase **(D)** positivities (Avidin-Biotin Complex immunostaining, scale bars included).

X-rays might be unremarkable in the early stage of the disease and this does not exclude the diagnosis. Lytic, poorly define or well defined lesions can be seen early with progressive sclerosis or cortical thickening of the bone in the reparative phase (4, 10, 55–59). We and others have suggested fluorodeoxyglucose (FDG), positron emission tomography (PET)-computed tomography (CT)-scan, and bone scintigraphy as diagnostic tools (6). However, this kind of imaging is not recommended for children because of the use of radiation. Bone scintigraphy has been replaced by whole body magnetic resonance imaging (MRI) to assess multifocal and extent of involvement, soft tissue involvement and exclude other diagnoses. The most typical findings include bone marrow edema (increased signal on T2 weighted MRI images and reduced signal on T1 weighted MRI images) and characterized by an enhancement and periostitis/periosteal reaction (**Figures 1**, **2**). Often associated with extensive soft tissue edema and inflammation. Joint involvement is sometimes seen with signs of synovitis on MRI. Three patterns have been described with the use of whole-body MRI: (1) Tibia-appendicular multifocal pattern with tibial lesions, multifocal and no clavicular involvement (**Figure 1**), (2) clavicular-spinal pauci-focal pattern: clavicular lesions and a few other predominantly spinal lesions without tibial involvement (**Figure 2**), and (3) Tibia-clavicular crossover pattern (60, 61). The long bone methaphysis, particularly clavicle (classic for CRMO)- and lower extremities (distal femur, proxima and distal tibia and distal fibular are most frequently affected (51, 62). Clavicle or mandible are the most common if a solitary site is involved, which is extremely rare.

## Pathology

A bone biopsy is a gold standard for diagnosis. Histologic sections of the bone include bony tissue showing areas of resorption with increased osteoclasts and cellular infiltrates, which may be highly dependent on the illness stage. In the beginning, there is a neutrophil predominance in the bone marrow cavities. An abscess can also be found, but Gram staining and other special stains fail to identify any microorganisms. Mast cell infiltrates have been described (9, 13, 63–66). Thus, the special stain tryptase and the immunohistochemical stain CD117 may be necessary for the bone pathology workup (**Figure 3**). In the late stages of CRMO, macrophages, lymphocytes, and plasma cells belong to the mixed inflammatory infiltrates often detected under the lens. Occasionally, noncaseating granulomas have been reported. In this setting, the differential diagnosis with sarcoidosis needs to be raised at this point. Ultimately, osteolysis, osteonecrosis, the recruitment of multinucleated giant cells, fibrosis, and, finally, sclerosis seem to be characteristically seen in the late stage of the disease. Of note, it is important to remember that the fibrosing end-stage may be detected already after a few years of active osteolysis with inflammation.

## Treatment

CRMO is a complex and complicated disease, and a multidisciplinary approach to treating this illness is critical. Some of these patients associate cutaneous and gastrointestinal symptoms to chronic bony pain. The goal is to relieve pain and prevent disease progression with the ultimate aim to annihilate the permanent damage in the affected bones. Non-steroidal anti-inflammatory drugs are the first line of treatment, but they are often insufficient to control the disease’s pain and progression. Therefore, biorepositories may need to be implemented for clinical trials (67). The institutions of worldwide accessible biorepositories are vital due to the rarity ad variability of CRMO. Randomized controlled trials have been difficult to execute in places where a laboratory information system is not fully operative (68). The CNO/CRMO subgroup of the Childhood Arthritis and Rheumatology Research Alliance recently produced a consensus treatment plan with three treatment arms (29). These include (1) Conventional disease-modifying antirheumatic drugs (DMARDs): methotrexate or sulfasalazine; (2) Biologic DMARDs: TNF-a inhibitors with or without concomitant methotrexate; and (3) Bisphosphonates. In all regimens, short courses of glucocorticoids are permitted. Among TNF-a inhibitors, infliximab has shown improvement in many CRMO patients as it is used in patients with inflammatory bowel disease (69–75). Etanercept has demonstrated superb clinical outcomes involving improvement in skin manifestations, and it is well tolerated. Adalimubab and certolizumab pegol have been demonstrated to be very successful. They show complete remission of skin and musculoskeletal manifestations. Other biologics include IL-1 blockers, IL-23, and IL-17 inhibitors with relatively promising results (11, 36, 54, 76–79). Bisphosphonates are effective and well-tolerated. In children, pamidronate is the bisphosphonate of choice.

## Prognosis

As indicated above, the outcome may be variable. Still, poor factors consist of prolonged time from symptoms to the final diagnosis, multifocality, and male gender (51). If treated early, CRMO may show a favorable outcome. Severe complications of this chronic bony disease include limb length discrepancy, deformity of the extremities, or vertebral fracture (4, 80). When matched with healthy children, children who were adequately diagnosed and treated did not demonstrate significant variations in objective amounts of physical activity and fitness (81).

## Conclusion

CRMO is a rare autoinflammatory disease of the skeleton with an impressive debilitating burden for skeletal and extra-skeletal systems. Despite numerous efforts to convey biorepositories to identify biomarkers, the diagnosis remains of exclusion. Two methods of diagnosis are not incompatible, and some of the criteria overlap. Nevertheless, bone biopsy remains the gold standard for diagnosing CRMO. Several lines of therapy exist with the TNF-α inhibitors showing effectiveness and good compliance.

## Author contributions

CS conceptualized the study. CS collected data, drafted the initial manuscript, and revised the manuscript. CS revised the data and performed the analysis, was responsible for the intramural funding, and revised the final draft of the manuscript. EM collected the imaging data, prepared the figures, and revised the manuscript. FS revised the manuscript and expanded on the genetics of the disorder. MZ revised the manuscript and was responsible for part of the funding. All authors meet the ICMJE requirements for authorship, approved the final manuscript as submitted and agree to be accountable for all aspects of the work.

## Funding

This research has been funded by the generosity of the Children's Hospital of Eastern Ontario, University of Ottawa, Ontario, Canada, and the Department of Orthopedics, Tianyou Hospital, Wuhan University of Science and Technology, Wuhan, Hubei, P.R. China. The funders had no role in study design, data collection, analysis, decision to publish, or manuscript preparation.

## Conflict of interest

The authors declare that the research was conducted in the absence of any commercial or financial relationships that could be construed as a potential conflict of interest.

## Publisher’s note

All claims expressed in this article are solely those of the authors and do not necessarily represent those of their affiliated organizations, or those of the publisher, the editors and the reviewers. Any product that may be evaluated in this article, or claim that may be made by its manufacturer, is not guaranteed or endorsed by the publisher.
